# Systematic Evaluation of Methods for Integration of Transcriptomic Data into Constraint-Based Models of Metabolism

**DOI:** 10.1371/journal.pcbi.1003580

**Published:** 2014-04-24

**Authors:** Daniel Machado, Markus Herrgård

**Affiliations:** The Novo Nordisk Foundation Center for Biosustainability, Technical University of Denmark, Horsholm, Denmark; The Pennsylvania State University, United States of America

## Abstract

Constraint-based models of metabolism are a widely used framework for predicting flux distributions in genome-scale biochemical networks. The number of published methods for integration of transcriptomic data into constraint-based models has been rapidly increasing. So far the predictive capability of these methods has not been critically evaluated and compared. This work presents a survey of recently published methods that use transcript levels to try to improve metabolic flux predictions either by generating flux distributions or by creating context-specific models. A subset of these methods is then systematically evaluated using published data from three different case studies in *E. coli* and *S. cerevisiae*. The flux predictions made by different methods using transcriptomic data are compared against experimentally determined extracellular and intracellular fluxes (from 13C-labeling data). The sensitivity of the results to method-specific parameters is also evaluated, as well as their robustness to noise in the data. The results show that none of the methods outperforms the others for all cases. Also, it is observed that for many conditions, the predictions obtained by simple flux balance analysis using growth maximization and parsimony criteria are as good or better than those obtained using methods that incorporate transcriptomic data. We further discuss the differences in the mathematical formulation of the methods, and their relation to the results we have obtained, as well as the connection to the underlying biological principles of metabolic regulation.

## Author Summary

Constraint-based modeling has become one of the most successful approaches for modeling large-scale biochemical networks. There are nowadays hundreds of genome-scale reconstructions of metabolic networks available for a wide variety of organisms ranging from bacteria to human cells. One of the limitations of the constraint-based approach is that it describes the cellular phenotype simply in terms of biochemical reaction rates, in a way that is disconnected from other biological processes such as genetic regulation. In order to overcome this limitation, different approaches for integration of gene expression data into constraint-based models have been developed during the past few years. However, all the methods developed so far have only been tested using isolated case studies. In this work, we elaborate a detailed survey of these methods, and perform a critical and quantitative evaluation of a selected subset of methods, using experimental datasets that include different organisms and conditions. This study highlights some of the current limitations in many of these methods, and reveals that no method published so far systematically outperforms the others.

## Introduction

During the past years, there have been successful applications of computational modeling of cellular metabolism in biotechnology [1], [2]. Different mathematical formalisms have been proposed for this goal, with kinetic and constraint-based models among the most widely adopted ones [3]. Whereas kinetic modeling requires extensive experimental data for determining the rate laws and kinetic parameters of biochemical reactions, constraint-based modeling mainly requires knowledge of the stoichiometry of the metabolic network. This information can be obtained from annotated genome sequences and metabolic pathway databases, making this approach quite suitable for the reconstruction of metabolic models at the genome scale. Constraint-based models describe the range of steady-state flux distributions of a metabolic network, using the so-called flux balance analysis (FBA) approach [4]. The simplicity and scalability of this approach, coupled with the advances in genome sequencing, has made constraint-based modeling a popular framework within the scientific community, and led to an explosion in the number of genome-scale metabolic reconstructions currently available [5]. These reconstructions range from microbes to higher organisms, and have been used for myriad applications, including the prediction of cellular phenotypes, design of microbial cell factories, studies of evolution, and model-driven discovery of novel drug targets [2], [6].

In order to study the effects of environmental perturbations or genetic manipulations on cellular metabolism, one can measure changes at the transcript, protein, metabolite, and flux levels. However, analyzing the coordinated behavior of the different biological processes requires the integration of this information under one common framework. Although flux measurements can be easily integrated into constraint-based models [7], there is no straightforward way to integrate other sources of data. For that reason, new methods to integrate different kinds of *omics* data into constraint-based models are being developed [8].

**Figure 1:**
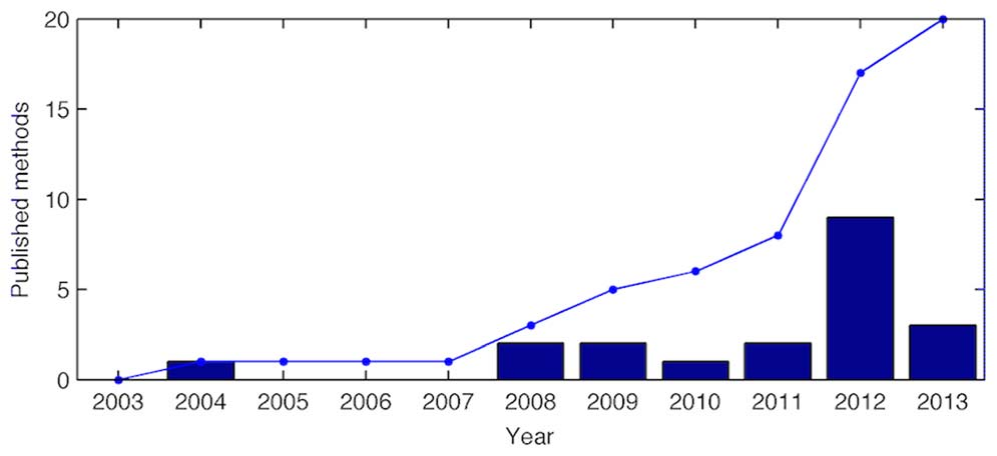
Publications per year. Number of publications of methods for integration of transcriptomic data into constraint-based metabolic models: publications per year (bars); cumulative sum (lines).

The advancements in high-throughput sequencing methods have increased the speed and decreased the cost of DNA and RNA sequencing [9]. Therefore, it is no surprise that most constraint-based methods for integration of experimental data have focused on the transcriptome. Some of these methods have already been covered in recent reviews [8], [10], [11]. However, they have not been critically and quantitatively evaluated using the same validation data. Furthermore, new methods are being developed at a fast pace (Fig. 1) and common method validation and comparison methodologies are needed to assess these methods.

Novel methods in computational biology are usually evaluated with dedicated case studies and data sets in the publications that introduce them. However, it is difficult to systematically evaluate and compare methods as the cases and data sets used in different publications are usually different. For this reason, there has been increased attention recently on performing dedicated studies that compare multiple methods using common cases and data sets [12]. Such studies can reveal differences in similar approaches and help in identifying strengths and weaknesses of particular approaches [13], [14]. These types of method comparison studies also play an important role in ensuring the reproducibility of computational research as they verify that published method implementations function as intended with novel data sets [15].

This work presents a comprehensive survey and a critical evaluation of the methods for integrating transcriptomics data to genome-scale metabolic models published thus far, with the purpose of guiding a careful methods selection and discussing limitations of the existing methods. In the spirit of reproducible research, we also aim to make it easier to evaluate new methods developed in the future by making all of our code and preprocessed datasets publicly available to the scientific community.

### Survey of methods

**Figure 2:**
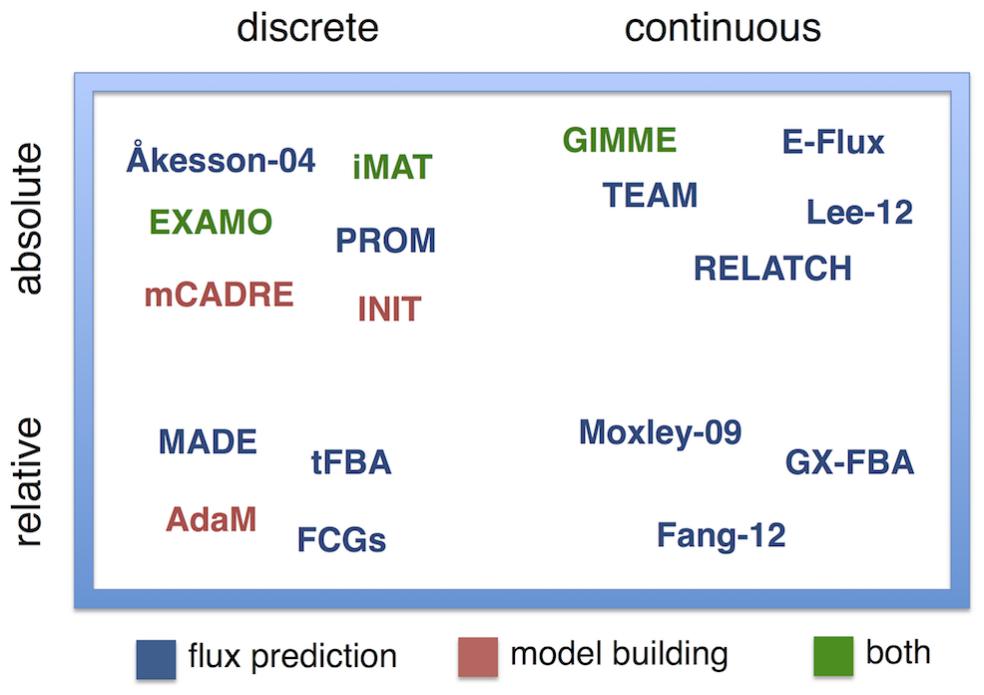
Methods overview. Classification of the methods regarding how they treat the gene expression levels (discrete *vs* continuous, absolute *vs* relative) and their intended functionality regarding flux prediction, model building or both.

The methods presented in this survey tend to fall into one of two categories. One encompasses all the methods that use transcript levels in order to improve the prediction of metabolic flux distributions. On the other hand are the methods for creating tissue (or context) specific models from more generic organism-specific models. A typical example is the creation of models for different kinds of human cells using the global human metabolic reconstruction, which can be used for the study of tissue specific diseases [16], [17]. Note that some methods fall into both categories, *i.e.* they return both a context-specific model and a metabolic flux distribution for the complete model consistent with the gene expression data. At the implementation level, the methods differ mainly in the way they use the expression data, by integrating either discrete or continuous expression levels, and by using absolute values for a single condition, or relative expression levels between different conditions (Fig. 2).

#### Åkesson-04 (2004)

One of the earliest approaches to integrate gene expression data into constraint-based models was proposed by Åkesson and co-workers with the purpose of improving flux predictions [18]. In this very simple approach, some reactions are deactivated, by constraining their flux to zero, if their associated genes are expressed at low levels. Using this method, the authors observed improved predictions in the metabolic flux distributions of *S. cerevisiae*.

#### GIMME (2008)

Gene Inactivity Moderated by Metabolism and Expression (GIMME), uses gene expression data to build context-specific models [19]. This method finds a flux distribution that is consistent with a given biological objective and that minimizes the utilization of reactions classified as inactive, weighted by the difference between their expression level and a given threshold. The authors used this method to model adaptive evolution in *E. coli* strains, and to create tissue-specific human cell models. A recent extension of the method, GIM3E, extends the previous method in order to force a minimum turnover rate for experimentally measured metabolites [20].

#### Shlomi–08/iMAT (2008)

The integrative metabolic analysis tool (iMAT) [16] implements a method proposed by Shlomi and coworkers for finding tissue-specific activity in the human metabolic reconstruction [21]. In this case, gene expression is used to divide reactions into two groups: highly and lowly expressed. It then finds a flux distribution that maximizes the consistency with this classification. It has the advantage of not requiring the definition of a biological objective, facilitating the analysis of biological systems, such as multi-cellular organisms, where this definition is not so clear.

#### Moxley–09 (2009)

Moxley and coworkers developed a method that predicts flux variation as a function of variation in gene expression [22]. It uses a pathway-specific parameter called “metabolite interaction density”, defined as the ratio of the number of metabolite-enzyme interactions to that of the total reaction enzymes in a pathway. Its calculation requires a topological reconstruction of all possible metabolite-enzyme interactions. The rationale for this method is that the correlation between changes in gene expression and reaction levels is likely to be higher in pathways with smaller interaction density, corresponding to increased degree of post-transcriptional regulation. The authors observed a significant increase in the correlation between measured and predicted fluxes after accounting for the interaction density parameter in their formulation.

#### E–Flux (2009)

E–Flux is a method that directly maps normalized gene expression levels into flux bound constraints using a “pipe capacity” analogy [23]. The rationale behind this method is that, although enzyme activities are not directly determined by their respective transcript levels, the latter can be used as an approximate upper bound for the reaction rates. In this case, the expression level of each gene is normalized by the maximum gene expression level across all genes. In a follow up study, the authors implement a similar formulation that, instead, normalizes each gene expression level by the maximum expression level of the same gene across multiple experiments [24].

#### PROM (2010)

Probabilistic regulation of metabolism (PROM) is a method for integration of regulatory and metabolic networks [25]. Given abundant gene expression data measured under multiple conditions, it generates a probabilistic model for the gene regulatory network, which is integrated with a constraint-based metabolic model by setting the flux bounds proportional to the associated probabilities. In principle, this approach can be used to integrate gene expression data directly into the metabolic model, using the fraction of times a gene is active in a set of samples. However, this requires an experimental data set with a large number of measurements per condition.

#### MADE (2011)

Metabolic Adjustment by Differential Expression (MADE) aims to overcome the problem of selecting arbitrary thresholds by comparing measurements across multiple conditions [26]. It uses the statistical significance between changes in gene expression levels across sequential conditions to find consistent series of activation/deactivation patterns. The solutions for all conditions are then solved simultaneously in order to maximize the agreement with the predicted patterns.

#### tFBA (2011)

Transcriptionally controlled FBA (tFBA) follows the same principles as MADE, albeit with a different formulation [27]. In this case, measurements across multiple conditions are also used, but they do not need to be from a time course. Up/down regulation events are included in the problem formulation, and the optimization problem consists of finding a suitable set of flux distributions across all conditions that minimizes the number of constraint violations.

#### INIT (2012)

The Integrative Network Inference for Tissues algorithm (INIT) is a method for building tissue-specific models from genome-scale reconstructions [28]. It was designed to use proteomic data from the Human Protein Atlas, but can also use transcriptomic data. It maximizes the activation of certain reactions based on a qualitative confidence score while minimizing the utilization of reactions associated with absent proteins. One of the novel aspects of this method is the relaxation of the steady-state condition to allow a small net accumulation rate for internal metabolites. If there is evidence for the presence of a metabolite, this accumulation is imposed in order to prevent the removal of the reactions necessary for its synthesis.

#### Lee–12 (2012)

Lee and coworkers proposed a method that integrates absolute gene expression data directly into the objective function of a constraint-based model, instead of manipulating the flux constraints [29]. The biological objective function is replaced by a function that minimizes the distance between the flux distribution and the gene expression data. The authors performed a comparison of this method against FBA, GIMME and iMAT, revealing a better accuracy in the prediction of secretion fluxes for *S. cerevisiae* under two growth conditions.

#### Fang–12 (2012)

The method developed by Fang and co–workers is based on the hypothesis that the flux distribution for a reference condition can be calculated with existing methods, and that the differential gene expression between a perturbed and reference condition can be used to predict the flux distribution for the perturbed state [30]. The method recalculates the flux distribution for the perturbed state, imposing flux bounds based on the relative gene expression between the two conditions. One of the novel aspects in this method is to allow small variations in the biomass composition for the perturbed condition, in order to account for the biomass variability that can occur under different growth conditions.

#### RELATCH (2012)

RELATive CHange (RELATCH) is a two-step method that uses flux and gene expression data from a reference state to predict metabolic responses in a genetically or environmentally perturbed state [31]. In a first step, transcriptomic and fluxomic data are used to predict a flux distribution and corresponding enzyme contributions for the reference state. The second step determines the flux distribution for a perturbed state that minimizes the adjustment to the reference distribution, and is consistent with the estimated enzyme contributions. Although this approach was designed to estimate flux distributions in perturbed states for which no expression data is available, the first step can, in principle, be used to predict flux distributions based on gene expression data for any given condition (we will refer to this variant as RELATCH*).

#### TEAM (2012)

Temporal Expression-based Analysis of Metabolism (TEAM) is a method that combines dynamic Flux Balance Analysis (dFBA) [32] and GIMME to predict time-course flux profiles based on temporal gene expression patterns [33]. It recalculates the flux distribution at each time step using the respective gene expression levels. It also offers some improvements over the original GIMME formulation, such as using gene-specific thresholds and using flux sum minimization to select among alternative optimal solutions.

#### AdaM (2012)

Adaptation of Metabolism (AdaM) is a method for integration of temporal gene expression data [34]. For each time point, it finds a minimal functional network consistent with the differential expression pattern. However, rather than calculating a flux distribution for each time point, it computes the set of elementary flux modes (EFMs) [35] for the subnetwork. The time-course pattern for each reaction is then represented by the fraction of EFMs containing the reaction at each time point.

#### GX–FBA (2012)

Gene-expression FBA (GX–FBA) is a flux prediction method that incorporates gene expression data into flux balance analysis [36]. It shares with E–Flux and Lee–12 the ability to directly use continuous expression levels. However, it uses differential gene expression between a perturbed and a reference condition rather than absolute expression values. It calculates a flux distribution for the reference condition, and then uses the relative expression levels to define flux constraints and a new objective function for the perturbed state.

#### mCADRE (2012)

Metabolic Context-specificity Assessed by Deterministic Reaction Evaluation (mCADRE) is a context-specific model building method [17]. Unlike similar methods such as the Model Building Algorithm (MBA) [37] that only uses transcript and protein data as evidence for metabolic functionality, mCADRE uses the gene expression levels and the network topology to calculate connectivity-based evidence scores for all reactions in a model. These scores are used to determine which reactions should be removed from the generic model to create a context-specific model.

#### FCGs (2013)

Flux-coupled genes (FCGs) is a method developed under the hypothesis that, although gene expression and flux levels are not always correlated, there might exist a subset of genes whose expression levels are consistent with the respective fluxes [38]. Since the identification of the FCGs requires transcriptome and fluxome data for the respective reactions under multiple conditions, the authors could only identify FCGs for the central carbon metabolism. Once identified, FCGs can be used to test new conditions by setting the upper bound of reactions associated with down-regulated FCGs to a fraction of the predicted flux. Furthermore, a sampling approach is applied in order to account for imperfect correlation between flux and gene expression.

#### EXAMO (2013)

The EXploration of Alternative Metabolic Optima (EXAMO) method for context-specific model building extends the iMAT approach by searching for multiple optima with the same agreement score [39]. The frequency of reactions in these multiple optima is calculated and used to build a context-specific model with MBA. A separate method is provided for flux estimation using the context-specific model, which minimizes the overall sum of fluxes while enforcing that a minimal flux is carried by the high frequency reactions. With this method the authors were able to predict the Crabtree effect in yeast cells growing in excess glucose.

## Results

In order to compare the predictive capability of the different methods, we tested their predictions using experimental datasets taken from the literature. From the initial survey of methods, we evaluated those that provide an implementation and that can be directly used for flux prediction. Note that although some of the methods not evaluated did provide an implementation, it was specific to a particular case study and not readily usable for general-purpose application.

In their original publications the methods have been validated using diverse sources of information and experimental data, usually not including actual fluxomics data (Table S1). Also, these publications rarely compare the proposed method with existing methods. The evaluation we performed requires a dataset with both transcriptomic and fluxomic data (ideally exchange rates as well as intracellular fluxes obtained by ^13^C-labeling) obtained using the same exact conditions for the same strain. However multi-*omics* studies that contain these two kinds of data are not common to find even for widely studied microbes such as *E. coli* and *S. cerevisiae*. A compilation of suitable multi-omic datasets is presented as supplementary material (Table S2). From these candidate datasets we selected the three that had intracellular flux profiles and transcriptome data: Ishii *et al* [40] and Holm *et al* [41] datasets for *E. coli*; and Rintala *et al* dataset for yeast [42].

In this study we attempt to address two main questions: First, how well can the methods predict the cellular physiology as well as the intracellular fluxes; Second, how does the integration of the measured physiological parameters (growth and secretion rates) influence the prediction of the intracellular flux distribution. Also, in order to understand how the integration of gene expression data can improve phenotype prediction, we compare the results with those obtained by standard FBA simulation. To avoid the typical degeneracy of FBA solutions, the parsimonious version of FBA (pFBA) was used. This approach finds a flux distribution with minimum absolute flux values among the alternative optima, assuming that the cell attempts to achieve its objective (here assumed to be maximization of biomass production) while allocating the minimum amount of resources.

### Case study: *E. coli* (Ishii)

This case study uses a comprehensive omics dataset published by Ishii *et al* [40]. The experimental setup consists of *E. coli* strains growing aerobically in a chemostat at a dilution rate of 0.2 h^−1^. The different experiments include variations of the dilution rate (from 0.1 to 0.7 h^−1^), and several single-gene knockout mutants growing at the reference dilution rate. For this dataset, the gene expression data is limited to the central carbon metabolism and is measured by microarray analysis.

**Figure 3:**
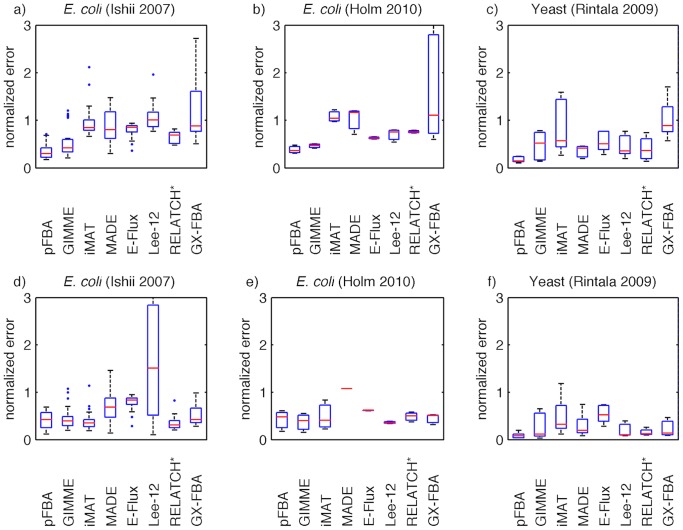
Prediction error for all methods. Distribution of the normalized prediction error for each method across multiple conditions for the different datasets. Each box plot represents the distribution of the prediction error for all conditions in one dataset. Two scenarios are evaluated: prediction of the complete metabolic phenotype (growth, secretion and intracellular fluxes) from measured uptake rates (a–c); and prediction of the intracellular fluxes from the measured physiology (growth, uptake and secretion rates) (d–f).

The assessed methods were applied to a genome-scale metabolic reconstruction of *E. coli* [43], to predict the complete phenotype (growth, secretion and intracellular fluxes) from the gene expression data, given only the measured glucose and oxygen uptake rates as constraints. Figure 3a shows the error distribution for the different methods (see Methods section for a description of the normalized error calculation). It can be observed that the median error for each method is higher than that of pFBA. Furthermore, many of the methods also have a higher variation in the error distribution compared to pFBA.

In order to understand how the phenotype predictions vary across the different methods, we analyze in detail a particular case, namely the experiment at the highest dilution rate (0.7 h^−1^). This is a typical case where FBA simulations are less accurate, since the assumption of growth yield maximization no longer holds true due to overflow metabolism. This is one of the experiments where pFBA gives a higher prediction error, and a likely scenario where alternative methods, such as those studied herein, will be most useful.

**Figure 4:**
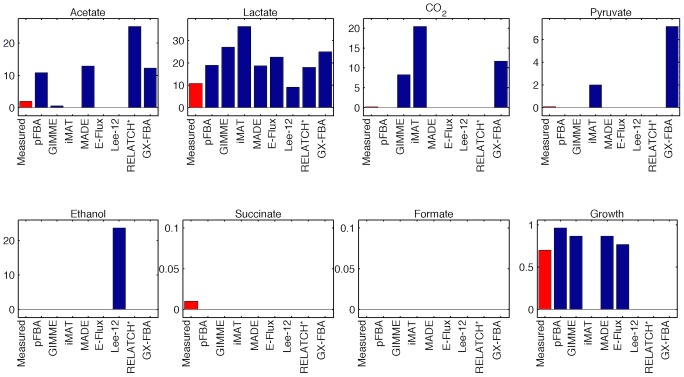
Physiology prediction (Ishii). Predicted and measured physiology: secretion rates (mmol/gDW/h) and growth rate (h^−1^), for the D = 0.7 h^−1^ experimental condition from the Ishii dataset.

The measured and predicted flux phenotypes are shown in Figure 4. It can be observed that, in most cases, the results differ significantly from the measured values. Since the oxygen uptake rate is constrained, pFBA is able to predict the secretion of fermentation products, namely lactate and acetate. However, it predicts higher values than the experimental ones. All the methods predict some level of lactate production, although not all were able to predict the production of acetate (iMAT, E–Flux, Lee–12). The residual amounts of CO2 and pyruvate produced were either not predicted by most of the methods, or overestimated by some methods (GIMME, iMAT, GX–FBA). Lee–12 incorrectly predicted a large production of ethanol. None of the methods predicted the production of succinate, and all correctly predicted the absence of formate production.

Regarding growth rate prediction, there are essentially two cases. The methods that maximize biomass production (pFBA, E–Flux) and the methods that impose some predefined threshold of biomass production (GIMME, MADE), predict values close to the maximum theoretical level. On the other hand, methods that do not impose any constraints regarding the growth rate simply predict no growth at all (iMAT, Lee–12, RELATCH*, GX–FBA).

In order to understand the influence of imposing experimental measurement constraints on the predictive ability of the methods, all the simulations were repeated using the complete set of measured uptake, growth and secretion rates as constraints (Fig. 3d). As expected, a decrease in the prediction error can be observed for many of the methods, with a higher impact on those that do not make assumptions regarding the growth rate (iMAT, RELATCH*, GX–FBA). On the other hand, Lee–12 exhibited an unexpected significant deterioration in performance when constraints were added.

A comparison between the predicted and measured fluxes across conditions for all the methods is given as supplementary material (Fig. S1). The experimental conditions are sorted by increasing error obtained by pFBA. Although there seems to be no correlation between the prediction errors across conditions, it can be observed that some of the methods exhibit a few biases towards systematically predicting higher or lower fluxes than experimental measurements for particular reactions.

Finally, we test whether integration of proteomic data (also included in this dataset) results in more accurate predictions than the use of gene expression data (Fig. S4). Despite some differences, there is no improvement in predictive ability when proteomics data is used instead of transcriptomics data.

### Case study: *E. coli* (Holm)

This case study uses a dataset from Holm *et al* [41], whose experimental setup consists of *E. coli* strains growing aerobically in batch cultures. The study compares the phenotype of the wild-type strain with two over-expression mutants, *nox* (NADH oxidase) and *atpAGD* (F1-ATPase), with the goal of understanding global transcriptional responses to lowered levels of NADH and ATP. The dataset contains gene expression data measured at the genome scale using microarray analysis and ^13^C-flux data.

The methods were tested using the same metabolic model as in the previous case study. In this dataset glucose uptake is the only measured uptake rate. The error distributions are shown in Figure 3b. Again, it can be observed that all methods show a higher median prediction error than pFBA. In this case, GX–FBA exhibits a much higher variation across conditions compared to the other methods.

**Figure 5:**
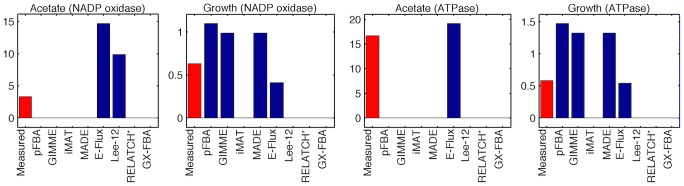
Physiology prediction (Holm). Predicted and measured physiology: acetate secretion rate (mmol/gDW/h) and growth rate (h^−1^) for the two over expression mutants (NADP oxidase and ATPase) from the Holm dataset.

The predicted phenotypes for the over-expression mutants are analyzed in more detail (Fig. 5). Unlike gene knockouts or gene insertions, over-expression targets do not change the topology of the metabolic network. Therefore, this is a typical case where the flux-balance formulation is insufficient to predict phenotypic changes. In fact, it can be observed that pFBA does not predict the decrease in growth rate and the increase in acetate secretion that characterizes these mutant strains. Only E–Flux was able to predict acetate production in both conditions, although in the first case the quantitative prediction is incorrect. As in the previous case study, only the methods that define a biomass objective or requirement predict positive growth rates. In this case E–Flux successfully predicted the growth rate to be below the theoretical maximum.

The impact of including the measured growth and secretion rates as constraints was also measured (Fig. 3f). As expected, most of the median error values decreased. Again, this impact is more significant for the methods that do not make any assumptions regarding the growth rate. A significant decrease in variation is observed for GX–FBA.

A comparison between the predicted and measured fluxes for all conditions is given as supplementary material (Fig. S2). It is interesting to observe that, especially in the cases of pFBA and E–Flux, the biases in flux prediction towards certain reactions are the same as observed in the previous case study.

### Case study: *S. cerevisiae*

This case study uses a dataset from Rintala *et al* [42], whose experimental setup consists of *S. cerevisiae* strains growing in a glucose-limited chemostat at a dilution rate of 0.1 h^−1^ with different oxygenation levels. These include intermediate levels from fully anaerobic to fully aerobic. The dataset contains genome-wide gene expression data. Fluxomic data for the same conditions could be obtained from a separate publication [44].

The assessed methods were used to integrate the gene expression data into a recent genome-scale metabolic reconstruction of *S. cerevisiae* [45]. Measured oxygen and glucose uptake rates were set as constraints. The error distribution for the different methods is shown in Figure 3c. As already observed in the previous case studies, all of the methods (with the exception of E–Flux) present a median prediction error above that of pFBA.

**Figure 6:**
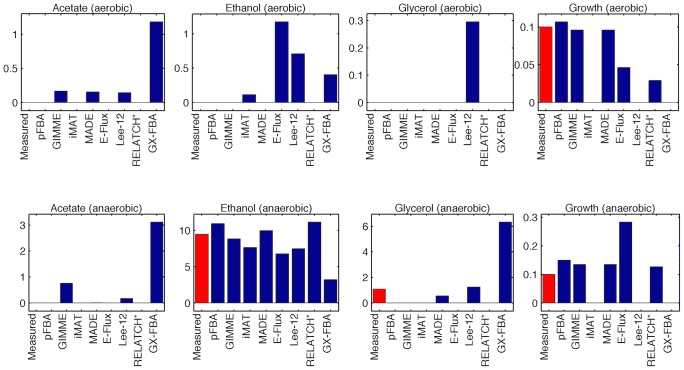
Physiology prediction (Rintala). Predicted and measured physiology: secretion rates (mmol/gDW/h) and growth rate (h^−1^) for two extreme conditions (full aerobiosis, full anaerobiosis) from the Rintala dataset.

We analyze in more detail the results for the two extreme conditions, complete aerobiosis and complete anaerobiosis (Fig. 6). For the aerobic case, the growth rate is very close to the maximum theoretical value, and no fermentation products are secreted. This is the typical case where the underlying assumptions of FBA are valid, as can be observed by the accuracy of the predictions. However, some of the methods incorrectly predict the secretion of some fermentation products. Under anaerobic conditions, the strain produces ethanol at high rates, and also a small amount of glycerol. All methods were able to predict ethanol production at rates similar to the experimental values, with the exception of GX–FBA that predicted a lower level of ethanol secretion accompanied with secretion of acetate and glycerol. GIMME and Lee–12 also incorrectly predicted the formation of acetate. On the other hand, Lee–12 predicted the glycerol secretion rate more accurately.

As in the previous case studies, we analyze the impact of including the complete physiological measurements (uptake, secretion and growth rates) as constraints (Fig. 3f). A decrease in the median prediction error is observed for most methods. Furthermore, a significant decrease in variability is observed for RELATCH* and GX–FBA. The comparison between the predicted and measured fluxes for all conditions is given as supplementary material (Fig. S3). In this case, very few systematic biases can be observed.

Finally, we tested whether integration of proteomic data (also included in this dataset) results in more accurate predictions than the use of gene expression data (Fig. S4). Since the number of transcripts whose levels could be measured is one order of magnitude above the number of proteins whose levels were measured, we recalculated the prediction error from transcript data using the subset of genes that match measured protein levels. Using only a subset of the transcriptomic data results in a small decrease in the variability of the prediction error, without affecting the median error. Furthermore, with the exception of E–Flux, there are no significant changes in the flux predictions when proteomics data is used instead of transcriptomics data.

### Sensitivity and robustness analysis

Three of the methods evaluated, namely GIMME, iMAT and MADE are parameterized, which makes the results presented so far dependent on the particular choice of the parameter configuration. Therefore, the sensitivity of the prediction error with respect to the parameter values was analyzed. For each case, one parameter was varied at a time while the others remained fixed (see Methods). In order to ensure that the results are not dependent on the case study, the analysis was performed for two datasets (Holm and Rintala).

The results show that for most parameters the variation is not monotonic with respect to the parameter value, and that the variance for one particular value can be larger than the average variation across the whole parameter range (Figs. S5, S6). Nevertheless, some trends can be observed. In general, higher cutoff thresholds for the gene expression data seem to be preferred, leading to the deactivation of more genes. A lower flux activation threshold is preferable for iMAT, and higher values of the required fraction of the biological objective seem to be favorable for GIMME and MADE. All these choices lead to parsimony in enzyme usage and maximization of the biological objective, which are the same principles used in pFBA. This is not surprising, considering that pFBA had in general better predictive power than other methods for all the case studies presented herein.

Finally, we tested the robustness of all the methods towards noise in the data (Fig. S7). The level of noise was gradually increased by a weighted combination of the original data with random data (see Methods). By gradually varying the noise weight from 0 to 1, the methods were given increasing levels of noise, including completely random data at the last step. This allows studying the robustness of the methods towards small levels of noise, as well as possible biases in the flux predictions in response to randomized data. The analysis was performed using the anaerobic condition from the Rintala dataset. This is a test case where all the methods have low error levels to begin with.

One would expect a smooth increase in the average prediction error with increasing noise level as an indicator of robustness. This increase in the error should also be accompanied by a gradual increase in the variance in flux predictions (made using different noisy transcript patterns generated at the same level of noise) as an indicator of the absence of systematic bias in flux predictions. However, only E–Flux exhibited this pattern. GIMME and Lee–12 show a gradual increase in the variance, although the average prediction error is the same for the original and the random data. MADE and iMAT show small changes in the average prediction error, coupled with a mostly constant level of variance. GX–FBA shows a smooth increase in the average prediction error, coupled with a sharp increase in variance, and fails to compute for very high levels of noise. RELATCH* shows an apparent constant level of the prediction error, with an increasing variation that is many orders of magnitude lower compared to the other methods. Hence the solution is biased regardless of the gene expression levels.

## Discussion

In this work we surveyed a wide variety of methods that integrate gene expression data into constraint-based models of metabolism. The publication rate of these types of methods seems to be rapidly increasing, which shows that the solution to this problem is far from trivial.

In general, these methods fall into one of two categories. They have been used to improve the prediction of metabolic flux distributions based on transcript levels, a useful tool for metabolic engineering of microbial cell factories [29], [31]. Also, they have been used to generate tissue (or context) specific models based on gene expression patterns, with potential applications in the study of multi-cellular organisms [16], [17], [28]. Since these are two distinct goals, we opted to focus this work on the evaluation of the former application. We believe that the latter application should be the subject of a dedicated study. As it was observed (see discussion below), the integration of transcript levels did not significantly improve flux predictions in a consistent manner. It is likely that the integration of gene expression data will prove itself more promising for the latter application. In the analysis of multi-cellular organisms, the biological principles commonly applied for microbes (such as maximization of growth rate) do not apply, and the definition of a tissue-specific objective is often unclear. Hence, the formulation of an objective function based on gene or protein expression data may provide a suitable alternative. Furthermore, a better correlation between gene expression and metabolic fluxes should be expected in multicellular organisms, due to the complete activation/deactivation of metabolic pathways in a tissue-specific fashion.

From the initial survey, seven methods were evaluated in detail, and their predictive ability was compared to that of FBA (in its parsimonious version). The experimental datasets used were selected in order to provide a variety of test scenarios, including a prokaryotic (*E. coli*) and an eukaryotic organism (*S. cerevisiae*). The experimental conditions included batch and chemostat fermentations, aerobic and anaerobic growth, as well as single gene deletions and over-expression mutants.

All the methods have a lower overall predictive capability compared to pFBA. At first sight this might indicate that the integration of gene expression data is hampering, rather than improving, the prediction of flux distributions. Given the variety of case studies it is unlikely that the selection of experiments was coincidentally favorable towards the pFBA approach. However, our evaluation could still be biased due to the fact that it is only possible to compare the simulated fluxes with intracellular flux measurements (from ^13^C-labeling) for the central carbon metabolism. It is known that the central metabolic pathways are more heavily regulated at post-transcriptional levels [46]–[48], hence transcript levels are in general not suitable for estimation of fluxes of the central carbon metabolism. It is likely that transcript profiles are better estimators of flux profiles of pathways that carry smaller fluxes. However, these fluxes will not be quantitatively correctly predicted if the central carbon flux profile is not correctly predicted as well.

In all the methods reviewed herein, transcript levels are used as surrogates for enzyme expression levels. Hence, if proteomics data are available, the latter could be used as well. In principle, protein levels should provide a more accurate snapshot of metabolism than transcript levels. However, we did not observe any improvements in the predictions when we used proteomic rather than transcriptomic data. This seems to indicate that the major obstacle in predicting fluxes from gene expression is the lack of correlation between protein levels and reaction rates.

There are multiple other possible reasons for the relatively poor predictive ability of many of the methods tested here. First, some of the methods were originally designed to make qualitative predictions and their ability to predict fluxes quantitatively was never assessed. Second, most methods do not try to incorporate other biological principles that may govern the cellular response such as minimization of overall flux magnitudes. It is known that in many cases transcriptional regulation acts as a mere modulatory factor in response to global cellular adjustments [49]. Finally, it is possible that some of the tested methods are better suited for particular conditions or organisms. Further studies could reveal why and when is gene expression a good predictor of metabolic flux distributions.

Since none of the methods evaluated in detail performs consistently better or worse than the other methods, we will not make any recommendations in favor or against any particular method. Users of these methods should perform a careful evaluation of the meaningfulness of the results for their particular applications. We further discuss in more detail some of the major differences found within the formulation of these methods, in order to guide the selection of a suitable subset of methods for a given application.

### Discrete *vs* continuous levels

One of the main features distinguishing the surveyed methods is the discretization of the gene expression data. It would seem preferable to make use of the continuous expression data in order not to lose the fine-grained data on the individual gene expression levels. Also, this avoids the definition of arbitrary threshold parameters. However, it is not possible to conclude that the methods that use continuous expression data (E–Flux, Lee–12, RELATCH*, GX–FBA) provide more accurate flux predictions than the ones that discretize the expression levels (GIMME, iMAT, MADE).

Discretization also presents a few advantages, such as robustness to noise in the data, seamless integration with the logic-based gene-protein-reaction (GPR) associations, and avoiding data normalization issues. Furthermore, coarse-graining the gene expression data reduces the reliance on a direct proportionality between the fluxes and the transcript levels.

### Absolute *vs* relative expression

Another major distinction between the surveyed methods is the choice between using absolute gene expression levels for one condition, or using differential gene expression between two or more conditions. One of the limitations of using absolute expression levels is the lack of proportionality between transcript and flux levels. A recent review from Hoppe highlights the multiple steps between gene expression and reaction rates [50]. Although some level of correlation can be observed between mRNA and protein levels, these are not directly proportional due to differences in translation, degradation rates, and post-translational modifications. Furthermore, enzyme concentrations do not necessarily reflect enzyme activity levels, as enzyme turnover numbers (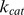) can vary by several orders of magnitude. Finally, metabolite concentrations, enzyme kinetics, and network level effects can influence the reaction flux as well.

Altogether it seems that enforcing a correspondence between absolute transcript and flux levels does not reflect the underlying biochemical mechanisms. In that sense, accounting for relative expression changes as an indicator of the intended flux reconfiguration may provide a more meaningful description. However, the methods that use relative expression levels (MADE and GX–FBA), did not generally give more accurate flux predictions.

### Biological objective formulation

Another distinction among the presented methods is the utilization of a biological objective function. The mathematical definition of a biological objective is the key step that transforms a metabolic network reconstruction into a model that can simulate the cellular phenotype. The maximization of growth yield, determined from the cellular biomass composition, has been a commonly assumed objective for microbial organisms. Although the validity of this assumption has been experimentally confirmed under some conditions [51], there are cases (such as overflow metabolism) where this assumption is not valid. Also, it has been shown that the biomass composition can vary across different experimental conditions [52]. Furthermore, in the case of multicellular organisms it is not trivial to define a biological objective.

All of the methods evaluated, with the exception of E–Flux, replace the biological objective function with a function that relies on the gene expression data. Nevertheless, some of these methods still use the original objective to define a minimum growth requirement constraint (GIMME, MADE) or to calculate a reference flux distribution (GX–FBA). Methods that do not make any assumptions regarding a biological objective (iMAT, Lee–12 and RELATCH*) should be suitable for a larger scope of organisms and experimental conditions. However, these methods incorrectly predicted a zero growth rate in all cases, with the exception of RELATCH* for the yeast case study.

In order to evaluate the effect of imposing a biological objective on all methods, we repeated all the tests, adding a minimum growth rate constraint, corresponding to 90% of the maximum theoretical growth rate, to all simulations (Fig. S8). We observed that the average error decreased for all the methods that do not impose any restrictions on the growth rate otherwise (iMAT, Lee–12, RELATCH*, GX–FBA). This decrease is similar to that observed by adding the experimental growth and secretion rates as constraints. Therefore, in the absence of experimental measurement, the imposition of constraints related to assumed cellular objectives may still be necessary for accurate flux predictions.

### Conclusions

Despite the high number of proposed methods, the prediction of flux levels from gene expression data is far from being solved. Although some of the methods evaluated give reasonable predictions under certain conditions, there is no universal method that performs well under all scenarios. Regardless of the mathematical formulation proposed to address the problem, the mapping of transcripts to fluxes is intrinsically hampered by the fact that gene expression levels do not necessarily reflect flux levels, which are systemic properties of the cellular metabolism. Nonetheless, the transcriptome should provide cues to guide the determination of the correct phenotype among the space of solutions that results from the large number of degrees of freedom in metabolic networks.

It has been proposed that the metabolic phenotype of microbial cells results from a trade-off between optimality and flexibility towards adaptation [53]. The optimality principles can be further decomposed into three distinct goals: growth yield, energy (ATP) yield, and parsimonious use of metabolic reactions. Hence, there are fewer inherent degrees of freedom in metabolism than the ones given by the network topology. Our study showed that growth yield and parsimony alone could be better predictors of metabolic fluxes than the transcriptome for most experimental sets. The ideal formulation to combine gene expression with fundamental biological principles governing metabolic flux distributions is yet to be found. This may require the integration of approaches that consider the interplay between transcripts and other metabolic components, by combining multiple omics data [20], [54] and kinetic parameters [55], [56] into constraint-based models. Alternatively, careful measurement of physiological parameters and intracellular fluxes coupled with separate analysis of transcript and flux patterns may be the most suitable strategy to uncover the principles of metabolic regulation [57]. These types of data can also be used to parameterize next generation of whole-cell models that explicitly represent proteins and transcripts in addition to metabolic fluxes [58], 

Finally, we would like to acknowledge the authors who published their source code with the respective articles. We would like to reiterate the importance of providing published methods in a usable format, a fundamental step for reproducible research [15]. With this in mind, all the scripts, datasets, and results generated from this work are freely available at: https://github.com/cdanielmachado/transcript2flux.

## Methods

### Model setup

The simulations for the *E. coli* and *S. cerevisiae* case studies were performed using, respectively, the iAF1260 and iTO977 genome-scale models [43], [45]. For all simulations, any constraints given in the original models were discarded and (depending on the test scenario) overridden with experimental values from the respective datasets.

### Methods setup

All method-specific configuration details are given in the following. All methods evaluated in this study have available implementations in MATLAB (The Mathworks; Natick, MA, USA). These were tested using MATLAB R2012b with Gurobi Optimizer 5.5 (Gurobi Optimization, Inc.) running on a 1.7 GHz Intel Core i5 processor.

#### pFBA

FBA simulations were performed using the available implementation in the COBRA Toolbox [60]. In all cases, the target objective was set to biomass production. To avoid the well-known degeneracy problem in FBA solutions [61] the option to minimize the Manhattan norm of the flux distribution was selected (according to the principle of parsimonious enzyme usage [62]). Note that when the growth rate is given as constraint, the result is simply the minimal flux distribution that complies with the imposed constraints. For simulation of gene deletions, the respective genes were deleted prior to simulation.

#### GIMME

The GIMME implementation in the COBRA Toolbox was used. However, this implementation discretizes the gene expression levels, which cannot then be used as weights in the objective function. This was changed to use continuous values as in the original publication [19]. GIMME takes two parameters: the gene expression cutoff value, which was set to the 25th percentile of the given expression data (this option was also adopted in the comparison done in [29]); and the required fraction of the original objective value, which was set to 90% of the maximum growth rate as in the original publication. Note that when the growth rate is given as a constraint, the latter parameter has no effect.

#### iMAT

The iMAT implementation in the COBRA Toolbox was used. However, this implementation does not use the tri-valued logic used in the original formulation. Therefore, this was changed to mapping scheme described in the original publication [21]. This method takes 3 parameters: the high and low expression thresholds, which were set to the 75th and 25th percentile of the given expression data (same as used in [29]); and the flux activation threshold, which was set to 1 as in the original publication.

#### MADE

An implementation of MADE is provided with the original publication. MADE integrates relative gene expression data for a series of sequential experiments into one large MILP formulation. This can create computationally intractable problems if the number of experiments is too large. Also, most datasets used in this study do not represent sequential experiments. Therefore, each experiment was individually coupled with a given reference condition. (Note that the requirement of sequential experiments is relaxed in a later implementation in the TIGER toolbox [63].) Similarly to GIMME, MADE also takes as parameter the required fraction of the original objective value, which was likewise set at 90%. If available, MADE can use p-values associated with gene expression changes to weight the respective objective coefficients. In this case, unit weighting was used.

#### E–Flux

Although the E–Flux publication does not offer an implementation, this method is simply an FBA problem with adjusted flux bounds. The rules for mapping gene expression to flux bounds were implemented as described in the original publication [23]. Note that the flux distribution obtained with E–Flux is adimensional. In order to compare it with experimental flux data, the resulting distributions were scaled by the given glucose uptake rates. For this method, exchange rate constraints are ignored, otherwise the solution space becomes infeasible.

#### Lee–12

An implementation for this method is provided with the original publication. Although the original formulation compares flux and gene expression values directly, in the implementation provided they are properly normalized to adimensional units. We followed the option taken by the authors to normalize the flux distribution by the glucose uptake rate, and the gene expression vector by the expression levels of the glucose transporters.

#### RELATCH

An implementation for RELATCH is provided with the original publication. This method provides two routines: the first uses gene expression data to calculate a flux distribution for a reference state; the second uses the reference state without additional expression data to calculate the flux distribution for a perturbed state. In this case, only the first routine was used (here referred to as RELATCH*). RELATCH uses the gene expression data directly in the objective formulation without any normalization. This makes the results dependent in the particular choice of units for the expression vector. Therefore, the expression vectors were divided by their mean prior to computation.

#### GX–FBA

An implementation for GX–FBA is provided with the original publication. This method uses flux variability analysis (FVA) [61] to calculate a reference flux distribution to be used for each perturbed state. Since this is a very computationally expensive step, it was pre-computed and passed as argument to all method invocations in order to speed up computation.

### Evaluation

#### Experimental data

Due to experimental error and differences in the models used in the ^13^C-labeling experiments, some of the experimental flux distributions do not lie precisely within the solution space of the genome-scale models used in this study. Since this would cause a systematic error in the evaluation of all methods, the experimental measurements were adjusted to the respective model by determining the feasible flux distribution with the smallest Euclidean distance to the original values.

#### GPR mapping

For the methods that use continuous gene expression, the expression levels were mapped from genes to reactions using the gene-protein-reaction (GPR) association rules in the models. In each case, the mapping was performed as described in the respective publication. In general, the expression level of reactions catalyzed by enzyme complexes (*and* operator) is set to the minimum expression level of the associated genes, and the expression level of reactions catalyzed by isozymes (*or* operator) is set to either the maximum or the sum of the expression levels of the associated genes. In order to deal with missing gene measurements, we opted to simplify the formula, removing the respective gene, rather than using an arbitrary expression value.

#### Removal of futile cycles

Most of the methods evaluated herein are prone to degenerate solutions due to the existence of futile cycles. This problem is avoided in the parsimonious version of FBA, but it is in general not avoidable in the other methods without altering their formulation. Given that this degeneracy can affect the evaluation results, all simulation results were post-processed in order to remove futile cycles. This done in a simple procedure where the set of reactions that participate in futile cycles are identified, and their absolute flux value is minimized, while the fluxes of all other reactions remain fixed.

#### Error measurement

For each simulation, the results were compared to the experimental measurements. The set of compared reactions include the intracellular fluxes for central carbon metabolism, the growth rate, and the secretion rates (the exact number of reactions can vary for the different datasets). The error of the estimation is given by the normalized Euclidean distance:
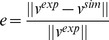
where 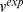 is the vector of flux measurements, and 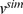 are the simulated values. This error metric is proportional to the summed square error (SSE) divided by the magnitude of the original flux vector, which facilitates the comparison across conditions. For a series of evaluations for the same method, the averaged error is given by the mean of the error for all experiments (failed computations are excluded).

#### Sensitivity analysis

Each parameter for GIMME, iMAT and MADE was varied independently, while the others remained fix at the default value described earlier. The required fraction of the biological objective for GIMME and MADE was varied linearly from 0 to 1. The flux activation threshold of iMAT varied from 0.1 to 10 in log-scale. The gene expression thresholds for GIMME and iMAT were defined in terms of the percentile of the given expression data, in order to make the results independent of the given units. The expression threshold for GIMME varied between the 0th and 100th percentile. For iMAT the low expression threshold varied from the 0th to the 75th percentile, whereas the high expression threshold varied from the 25th to the 100th percentile. For each range, a total of 20 equally spaced values were evaluated for the multiple experiments on each dataset.

#### Robustness analysis

All the methods were analyzed regarding their robustness to noise in the data. The noisy data was generated as follows. For a given experimental condition, the gene expression vector (*x*) was randomly shuffled in order to generate a random expression vector (*r*) that follows the same distribution as the original data. The noisy expression data (*y*) is given by:
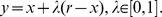
By varying the *λ* parameter it is possible to vary between intermediate noise levels that range from the original data (*λ* = 0) to completely random data (*λ* = 1). For each test, *λ* is varied by 10 equally spaced steps and, for each step, a total of 100 evaluations are performed and the average prediction error is measured.

## Supporting Information

Figure S1 **Individual flux predictions (Ishii).** Difference between predicted and measured fluxes (mmol/gDW/h) for all the evaluated methods, across all conditions from the Ishii dataset for *E. coli*. All the conditions are sorted by increasing error of pFBA simulation. The error distribution is individually scaled for each method. Missing columns represent failed computations.
(TIF)

Figure S2 **Individual flux predictions (Holm).** Difference between predicted and measured fluxes (mmol/gDW/h) for all the evaluated methods, across all conditions from the Holm dataset for *E. coli*. All the conditions are sorted by increasing error of pFBA simulation. The error distribution is individually scaled for each method. Missing columns represent failed computations.
(TIF)

Figure S3 **Individual flux predictions (Rintala).** Difference between predicted and measured fluxes (mmol/gDW/h) for all the evaluated methods, across all conditions from the Rintala dataset for *S. cerevisiae*. All the conditions are sorted by increasing error of pFBA simulation. The error distribution is individually scaled for each method. Missing columns represent failed computations.
(TIF)

Figure S4 **Transcriptomics** ***vs*** **proteomics.** Comparison of the normalized prediction error for each method across multiple conditions using either transcriptomic or proteomic data. Two scenarios are evaluated: prediction of the complete metabolic phenotype (growth, secretion and intracellular fluxes) from measured uptake rates (a–d); and prediction of the intracellular fluxes from the measured physiology (growth, uptake and secretion rates) (e–h).
(TIF)

Figure S5 **Sensitivity analysis (Holm).** Sensitivity analysis of the parameterized methods using the Holm dataset. The averaged normalized prediction errors across all conditions are presented.
(TIF)

Figure S6 **Sensitivity analysis (Rintala).** Sensitivity analysis of the parameterized methods using the Holm dataset. The averaged normalized prediction errors across all conditions are presented.
(TIF)

Figure S7 **Robustness analysis (Rintala).** Robustness analysis of the different methods towards increasing levels of noise in the data. The noise level varies from 0 (original data) to 1 (completely random data). Analysis performed using the anaerobic condition from the Rintala dataset.
(TIF)

Figure S8 **Comparison of the prediction errors for the original formulation of the methods (a–c) to the scenario where a minimum growth rate of 90% is enforced for all methods (d–f).** The absence of results for iMAT in the Holm dataset results from infeasibility of solutions using the imposed constraints.
(TIF)

Table S1 **Original methods validation.** Description of the validation approach and data sources for each method in their original publication.
(PDF)

Table S2 **Compilation of multiomics dataset references.** Compilation of literature references containing multi-omics datasets including transcriptomic and fluxomic data for *E. coli* and the yeast *S. cerevisiae*. The datasets used in this study are highlighted.
(PDF)
