# Correction: Systematic Evaluation of Methods for Integration of Transcriptomic Data into Constraint-Based Models of Metabolism

**DOI:** 10.1371/journal.pcbi.1003989

**Published:** 2014-10-27

**Authors:** The PLOS Computational Biology Staff

There is a labeling error in the metabolites of Figure 4, the updated figure is attached.

**Figure 4:**
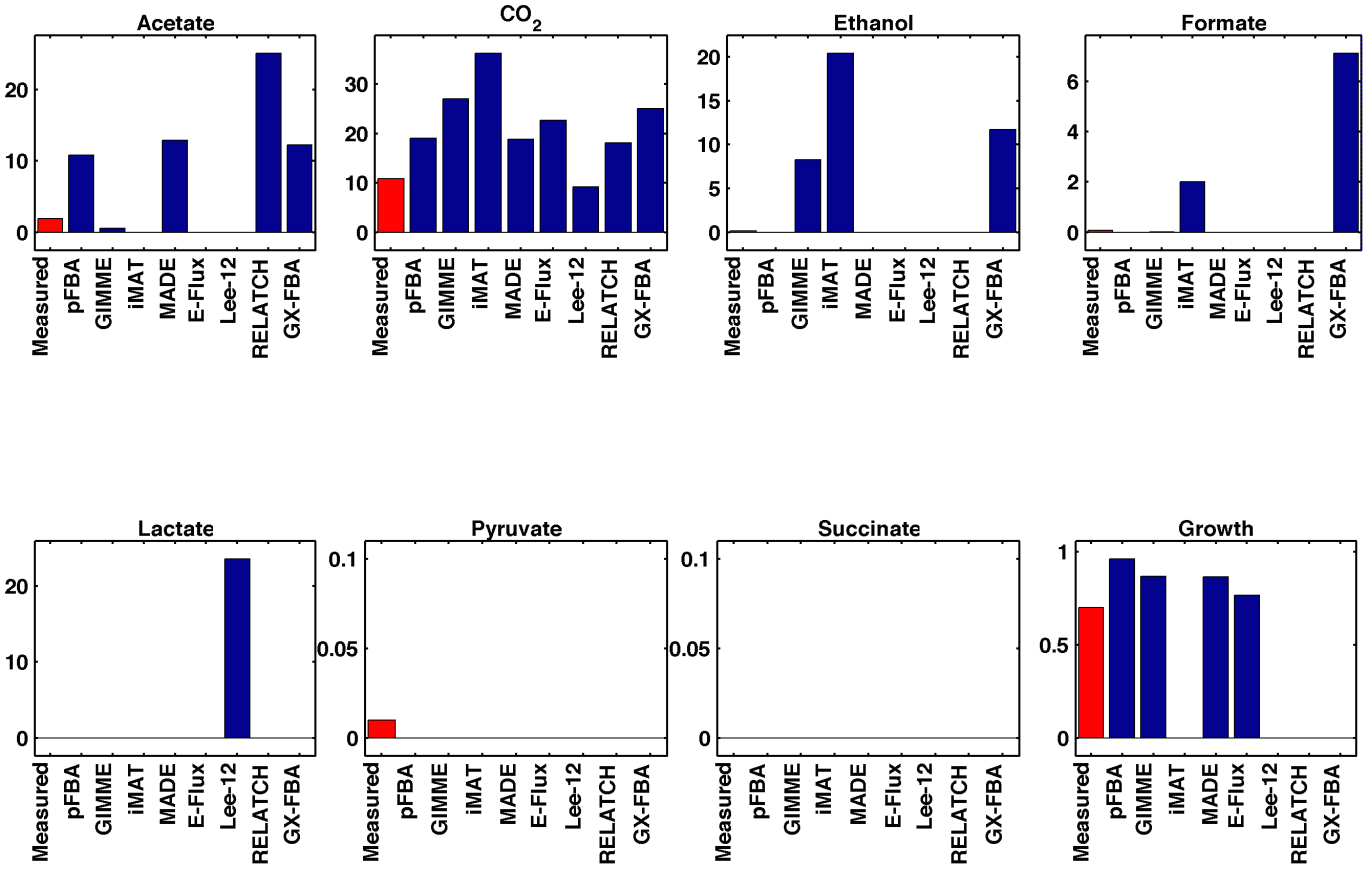
Physiology prediction (Ishii). Predicted and measured physiology: secretion rates (mmol/gDW/h) and growth rate (h^−1^), for the D=0.7 h^−1^ experimental condition from the Ishii dataset.

The fourth paragraph in the sub-section ‘Case study: E. coli (Ishii)’ of the Results section is incorrect. The paragraph should read:

“The measured and predicted flux phenotypes are shown in Figure 4. It can be observed that, in most cases, the results differ significantly from the measured values. Since the oxygen uptake rate is constrained, pFBA is able to predict the secretion of acetate. However, it predicts higher values than the experimental ones. Most methods predict the production of acetate with the exception of iMAT, E–Flux, and Lee–12. The residual amounts of ethanol and formate produced were either not predicted by most of the methods, or overestimated by some methods (GIMME, iMAT, GX–FBA). Lee–12 incorrectly predicted a large production of lactate. None of the methods predicted the residual production of pyruvate, and all correctly predicted the absence of succinate production.”

The authors would like to thank Matt DeJongh and Shinnosuke Kondo for detecting this error.
