# Supplementary figures and images for: Systematic Evaluation of Methods for Integration of Transcriptomic Data into Constraint-Based Models of Metabolism
Source: PLoS Comput Biol. 2014 Apr 24;10(4):e1003580. doi: 10.1371/journal.pcbi.1003580 (PMC3998872; doi:10.1371/journal.pcbi.1003580)

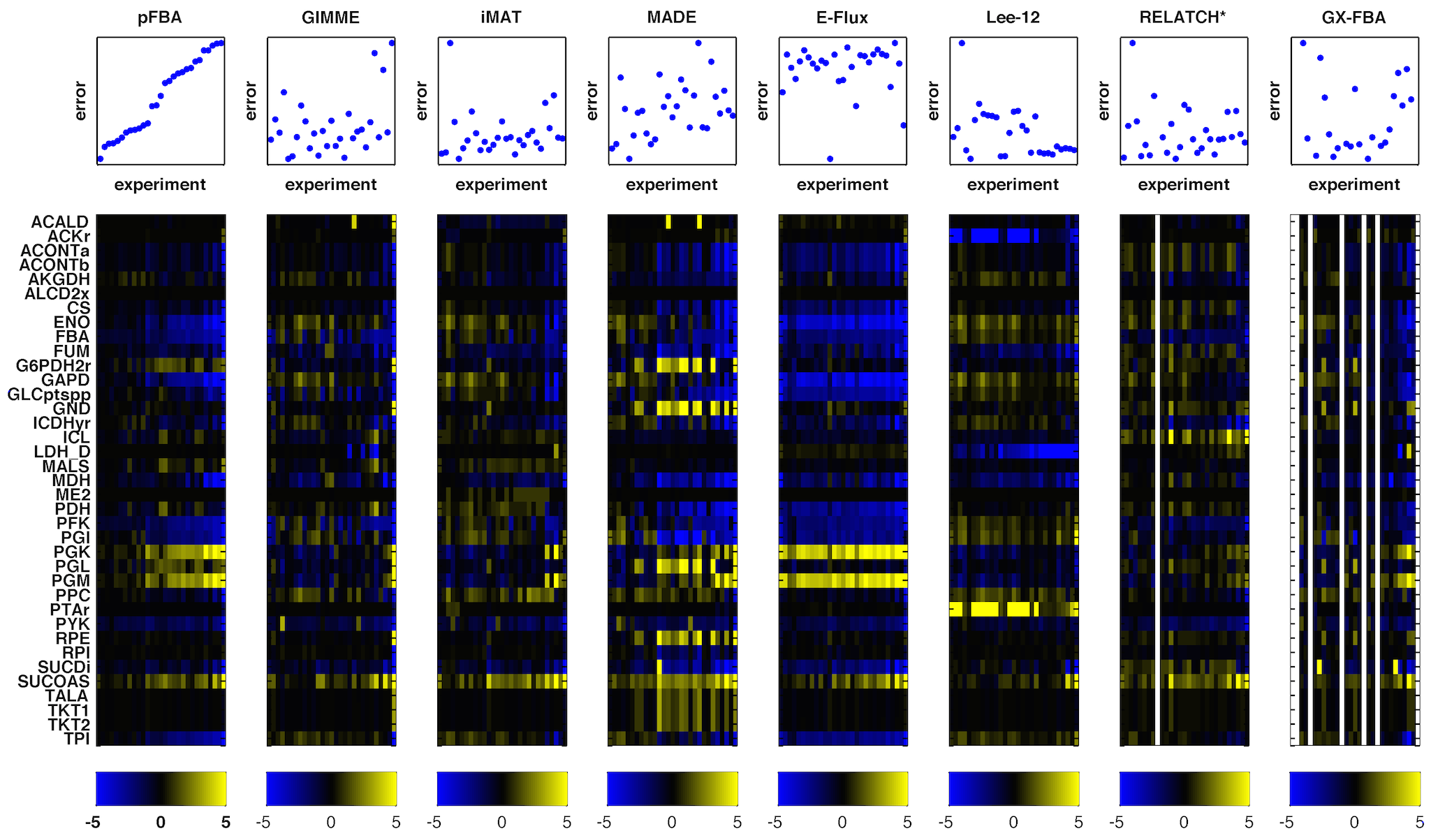

Supplement: Figure S1 — Individual flux predictions (Ishii). Difference between predicted and measured fluxes (mmol/gDW/h) for all the evaluated methods, across all conditions from the Ishii dataset for E. coli. All the conditions are sorted by increasing error of pFBA simulation. The error distribution is individually scaled for each method. Missing columns represent failed computations. (TIF) [file pcbi.1003580.s001.tif]

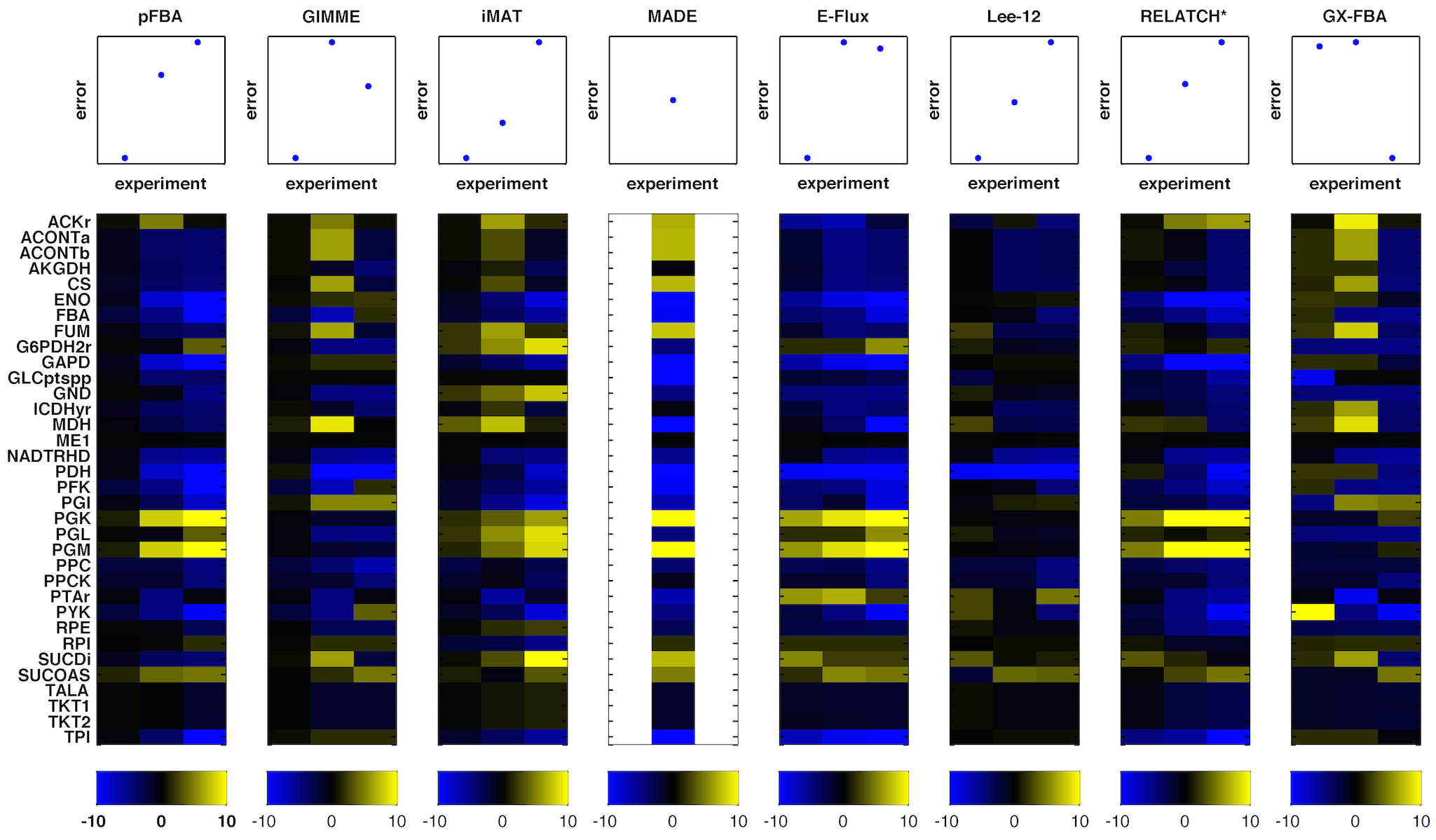

Supplement: Figure S2 — Individual flux predictions (Holm). Difference between predicted and measured fluxes (mmol/gDW/h) for all the evaluated methods, across all conditions from the Holm dataset for E. coli. All the conditions are sorted by increasing error of pFBA simulation. The error distribution is individually scaled for each method. Missing columns represent failed computations. (TIF) [file pcbi.1003580.s002.tif]

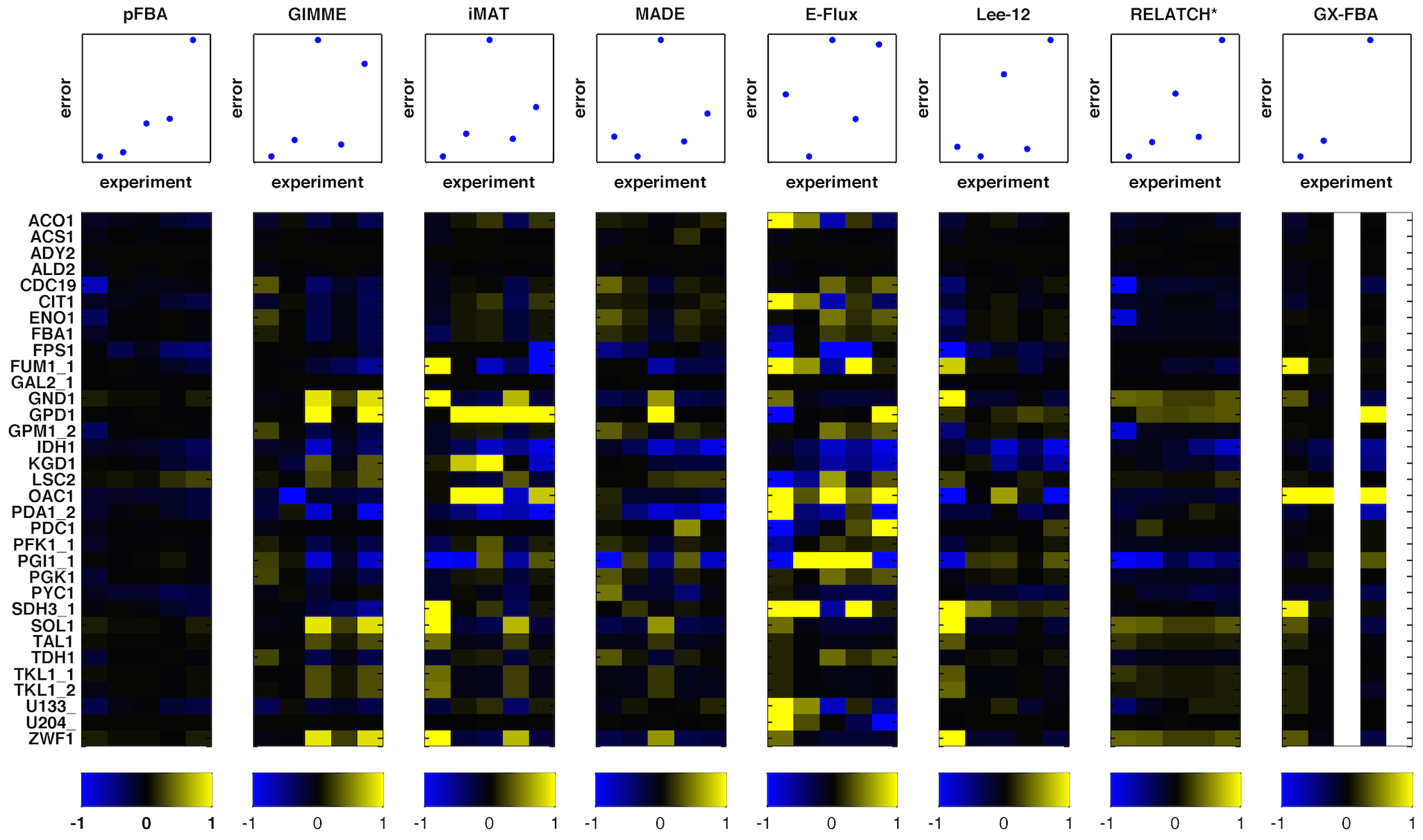

Supplement: Figure S3 — Individual flux predictions (Rintala). Difference between predicted and measured fluxes (mmol/gDW/h) for all the evaluated methods, across all conditions from the Rintala dataset for S. cerevisiae. All the conditions are sorted by increasing error of pFBA simulation. The error distribution is individually scaled for each method. Missing columns represent failed computations. (TIF) [file pcbi.1003580.s003.tif]

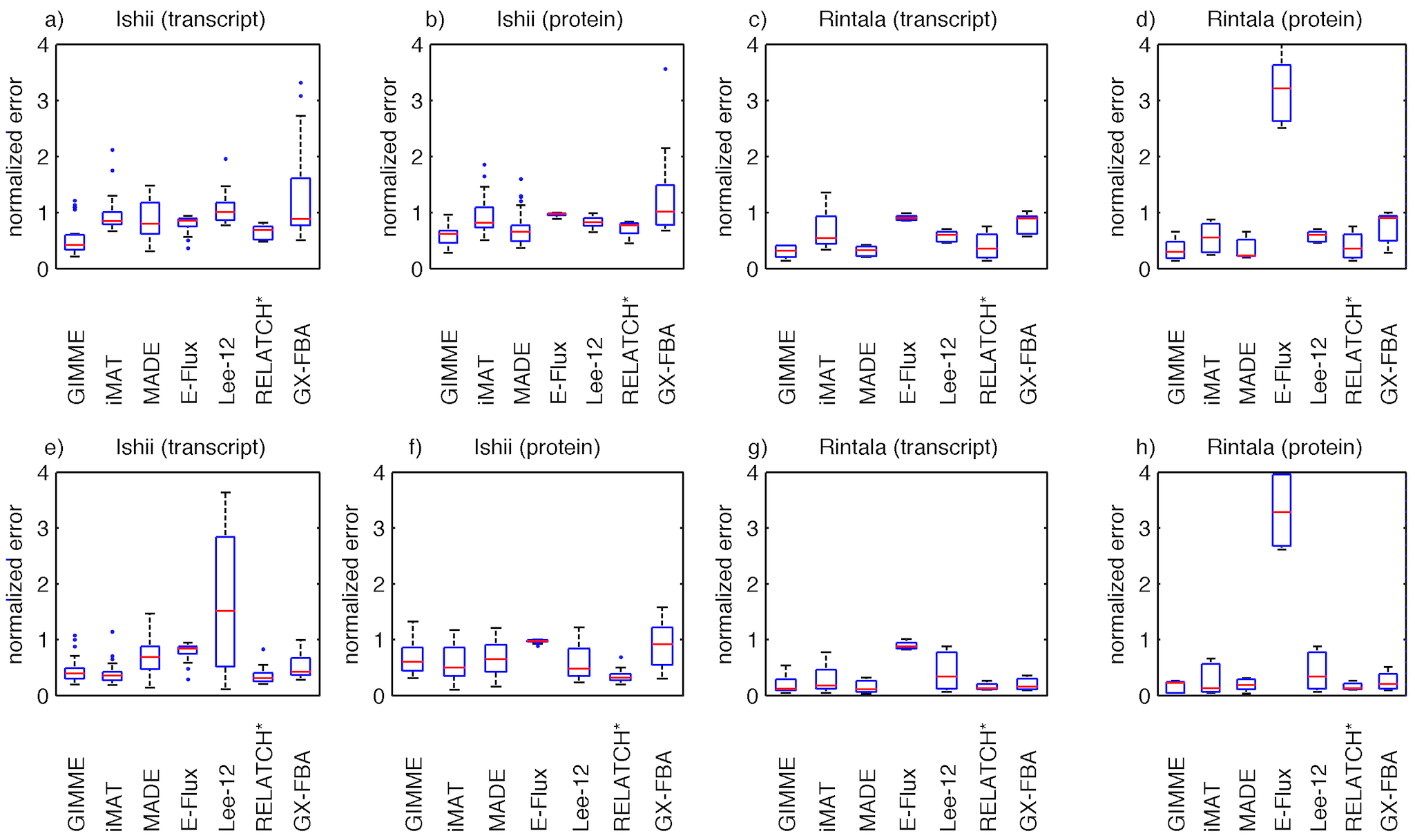

Supplement: Figure S4 — Transcriptomics vs proteomics. Comparison of the normalized prediction error for each method across multiple conditions using either transcriptomic or proteomic data. Two scenarios are evaluated: prediction of the complete metabolic phenotype (growth, secretion and intracellular fluxes) from measured uptake rates (a–d); and prediction of the intracellular fluxes from the measured physiology (growth, uptake and secretion rates) (e–h). (TIF) [file pcbi.1003580.s004.tif]

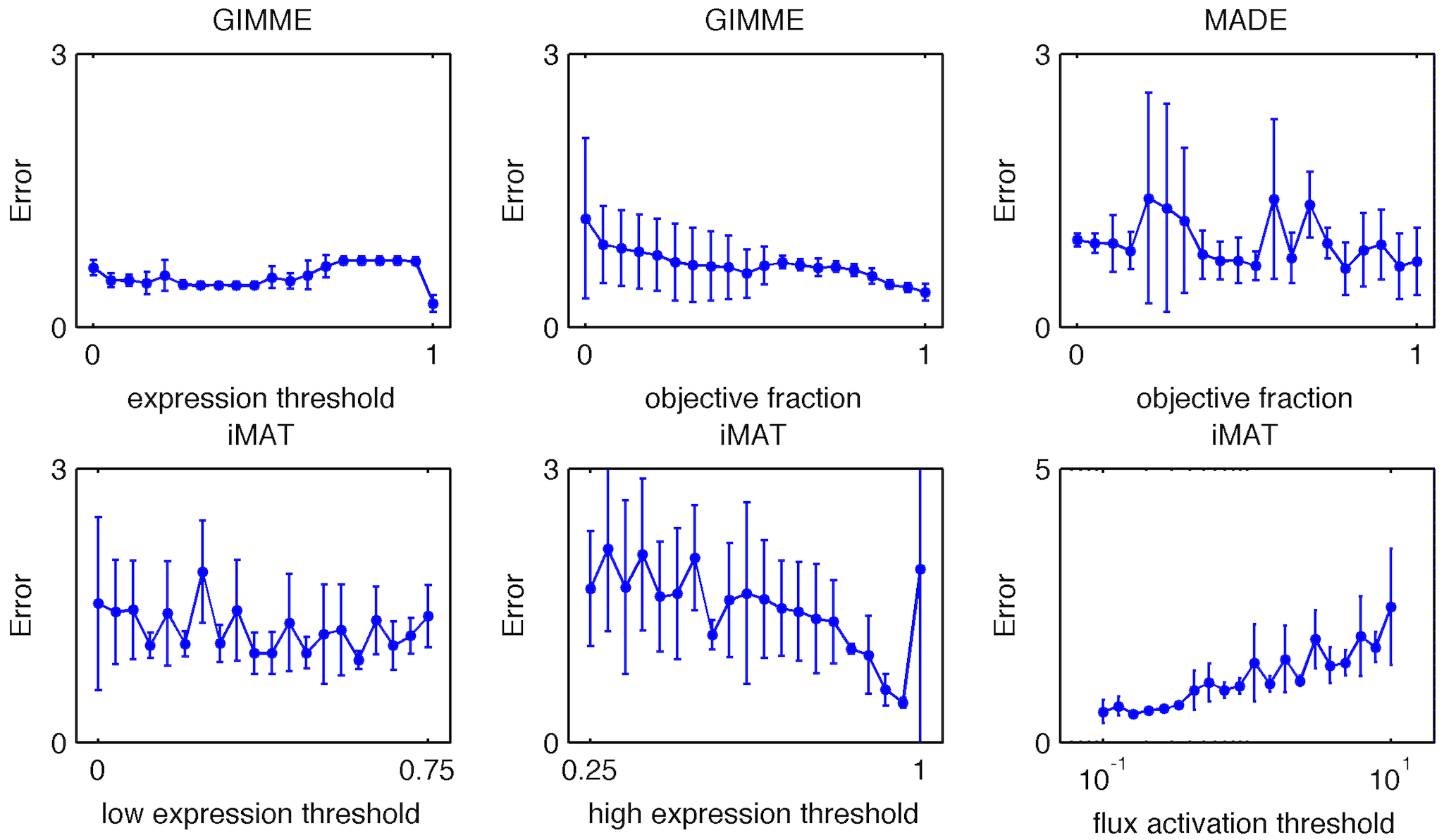

Supplement: Figure S5 — Sensitivity analysis (Holm). Sensitivity analysis of the parameterized methods using the Holm dataset. The averaged normalized prediction errors across all conditions are presented. (TIF) [file pcbi.1003580.s005.tif]

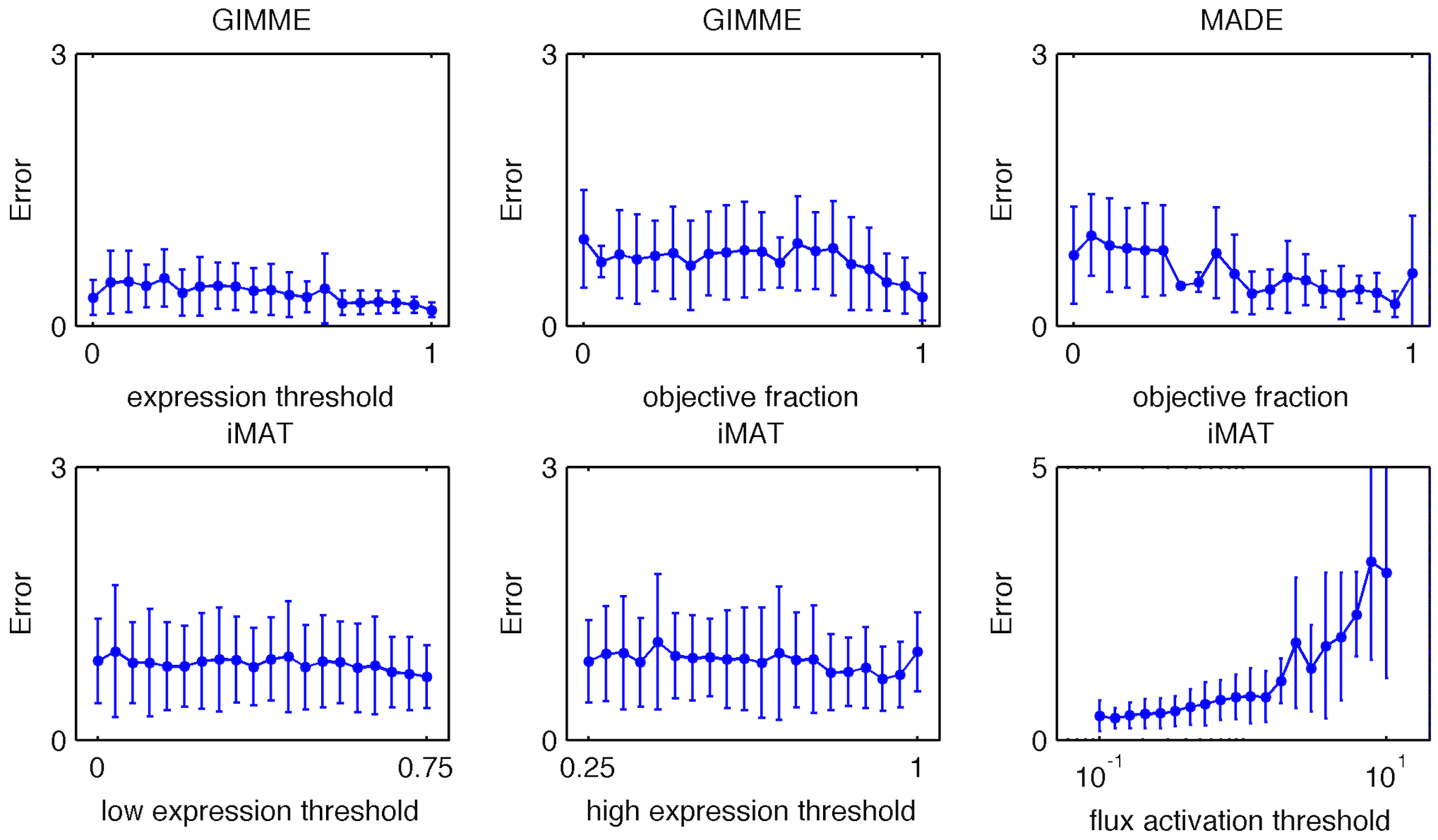

Supplement: Figure S6 — Sensitivity analysis (Rintala). Sensitivity analysis of the parameterized methods using the Holm dataset. The averaged normalized prediction errors across all conditions are presented. (TIF) [file pcbi.1003580.s006.tif]

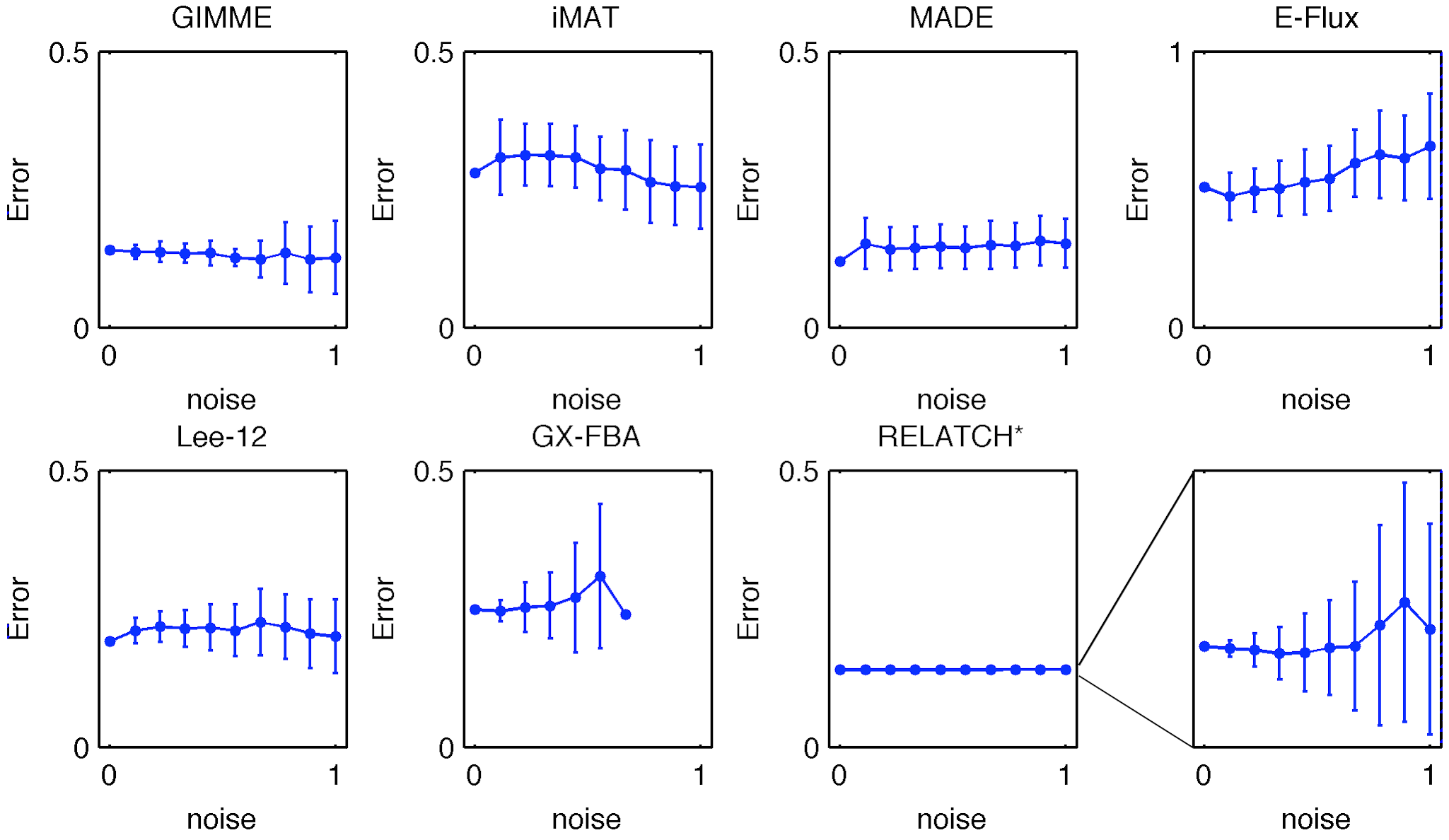

Supplement: Figure S7 — Robustness analysis (Rintala). Robustness analysis of the different methods towards increasing levels of noise in the data. The noise level varies from 0 (original data) to 1 (completely random data). Analysis performed using the anaerobic condition from the Rintala dataset. (TIF) [file pcbi.1003580.s007.tif]

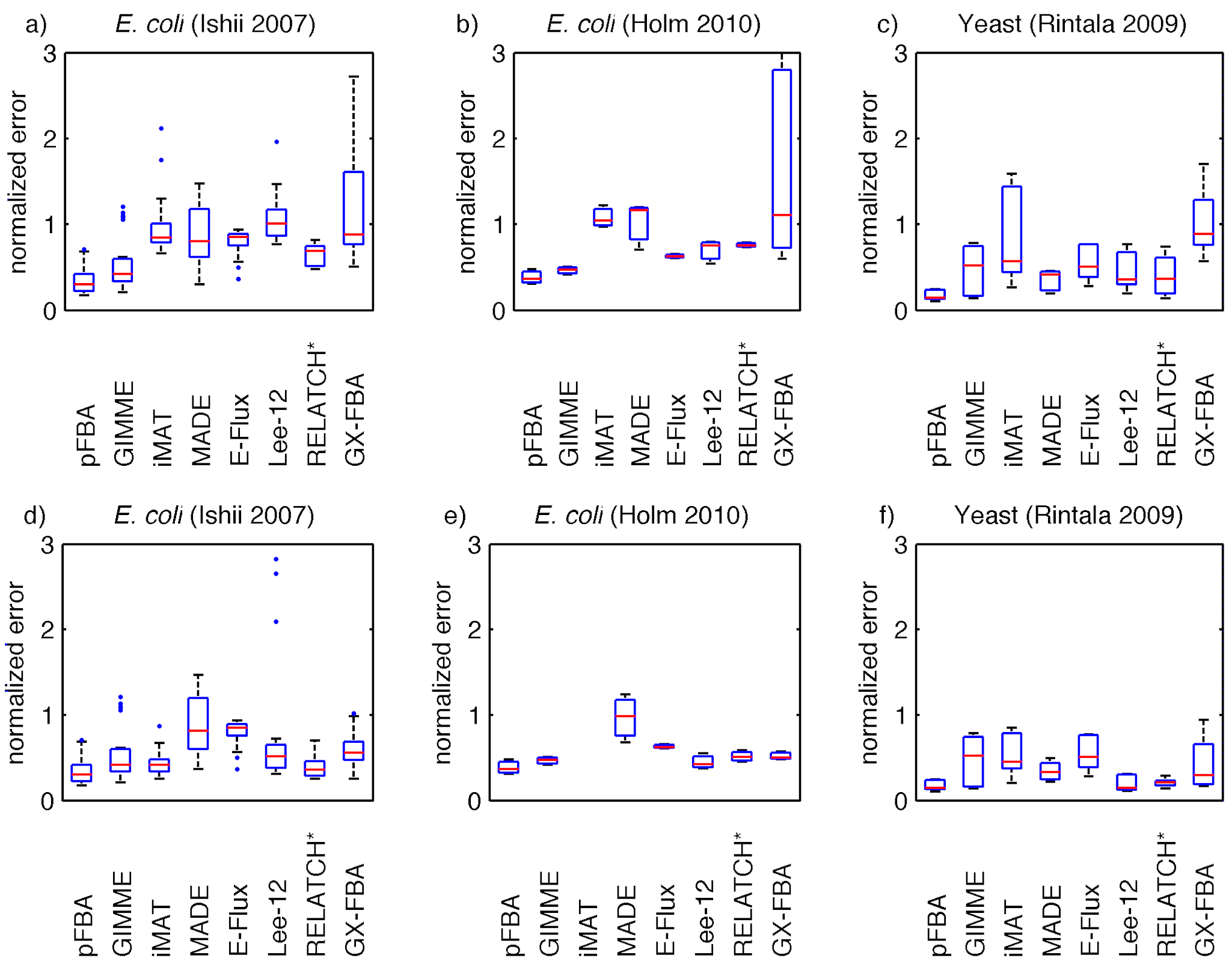

Supplement: Figure S8 — Comparison of the prediction errors for the original formulation of the methods (a–c) to the scenario where a minimum growth rate of 90% is enforced for all methods (d–f). The absence of results for iMAT in the Holm dataset results from infeasibility of solutions using the imposed constraints. (TIF) [file pcbi.1003580.s008.tif]
