# Supplementary material for: Systematic Evaluation of Methods for Integration of Transcriptomic Data into Constraint-Based Models of Metabolism
Source: PLoS Comput Biol. 2014 Apr 24;10(4):e1003580. doi: 10.1371/journal.pcbi.1003580 (PMC3998872; doi:10.1371/journal.pcbi.1003580)
Supplement: Table S1 — Original methods validation. Description of the validation approach and data sources for each method in their original publication. (PDF) [file pcbi.1003580.s009.pdf]

Method	Organisms	Input data	Validation	Methods compared
Akesson-04	S.cerevisiae	Piper et al, 2002	Secretion rates (same study) and 13C fluxes (Gombert et al, 2001)	
GIMME	E.coli, Human	Fong and Palsson 2004; Park et al, 2006; Parikh et al 2007	Consistency between original and readjusted expression levels	
iMAT	S.cerevisiae, Human	Lapujade et al, 2004; Shmueli et al, 2003; Mishra et al, 2006	Consistency of human tissue-specific gene activity with known evidence (multiple databases); S.cerevisiae MFA data (Lapujade et al, 2004)	
Moxley-09	S.cerevisiae	Same study	13C fluxes (same study)	
E-Flux	M.tuberculosis	Boshoff et al, 2004	Qualitative agreement with known inhibitory drugs (Boshoff et al, 2004)	
PROM	E.coli, M.tuberculosis	Covert et al 2004; Boshoff et al, 2004	Growth rate of multiple mutants for E.coli (Covert et al, 2004) and M.tuberculosis (Sassetti et al, 2003; Gao et al, 2005; Lamichhane et al, 2003)	rFBA
MADE	S.cerevisiae	Roberts and Hudson, 2006	Consistency between original and adjusted gene expression patterns	
tFBA	S.cerevisiae	Knijnenburg et al, 2009	Qualitative agreement between gene expression and flux changes (Boer et al, 2003; Tai et al, 2005)	FBA, rFBA
INIT	Human	Human Proteome Atlas database	Comparison of generated hepatocyte model with manually curated model, qualitative agreement of predicted drug targets in cancer cell lines	
Lee-12	S.cerevisiae	Same study	Secretion rates (same study)	FBA, GIMME, iMAT
Fang-12	S.cerevisiae, M.tuberculosis	Park et al, 2003	MFA data for S. cerevisiae (Lapujade et al, 2004). Qualitative agreement with known metabolic adaptation responses of M. tuberculosis	
RELATCH	E.coli, S.cerevisiae, B.subtilis	Covert et al 2004; Canelas et al, 2010; Tannler et al, 2008	13C fluxes for E.coli (Fong et al, 2006; Ishii et al, 2004), S. cerevisiae (Blank et al, 2005) and B.subtilis (Fischer and Sauer, 2005)	FBA, MOMA, ROOM
TEAM	S.oneidensis	Beg et al, 2012	Qualitative agreeement with temporal exometabome profiles (Beg et al, 2012)	dFBA
AdaM	E.coli	Jozefczuk et al, 2010	No validation with independent experimental data	MADE
GX-FBA	S.cerevisiae, Y.pestis	Lapujade et al, 2004; Han et al, 2004; Motin et al, 2004; Qiu et al 2005; Qiu et al 2006	MFA data for S.cerevisiae (Lapujade et al, 2004), qualitative agreement with known metabolic responses of Y.pestis	
mCADRE	Human	McCall et al, 2011	Qualitative agreement of generated models for liver and other tissues with literature based evidence	MBA
FCGs	E.coli	Ishii et al, 2007	13C fluxes (Ishii et al, 2007)	FBA, MOMA
EXAMO	S.cerevisiae	Gasch et al, 2000	Gene essentiality data (Giaever et al, 2002; Snitkin et al 2008); Predicted fluxes in qualitative agreement with known yeast physiology	MBA, iMAT
