# Supplementary material for: Systematic Evaluation of Methods for Integration of Transcriptomic Data into Constraint-Based Models of Metabolism
Source: PLoS Comput Biol. 2014 Apr 24;10(4):e1003580. doi: 10.1371/journal.pcbi.1003580 (PMC3998872; doi:10.1371/journal.pcbi.1003580)
Supplement: Table S2 — Compilation of multiomics dataset references. Compilation of literature references containing multi-omics datasets including transcriptomic and fluxomic data for E. coli and the yeast S. cerevisiae. The datasets used in this study are highlighted. (PDF) [file pcbi.1003580.s010.pdf]

| Organism | Transcriptomics | Fluxoxomics | Other data | Conditions | Reference |
| --- | --- | --- | --- | --- | --- |
| E. coli | genome wide | exchange (8) | | Batch growth of wild-type and 6 gene deletion mutants under aerobic and anaerobic conditions | Covert et al, 2004 |
| E. coli | central carbon (73) (relative expression) | internal (25) | | Batch growth (aerobic) of wild-type and 3 gene deletion mutants | Fong et al, 2006 |
| E. coli | central carbon (85) | internal (42) exchange (9) | proteomics metabolomics | Chemostat growth (aerobic) of wild-type at 5 dilution rates and 24 single gene deletion mutants (0.2 $h^{-1}$) | Ishii et al, 2007 |
| E. coli | genome wide | internal (25) exchange (2) | metabolomics | Batch growth (aerobic) of wild-type and 2 over expression mutants | Holm et al, 2009 |
| E. coli | genome wide (relative expression) | internal (32) exchange (8) | metabolomics | Chemostat growth (0.2 $h^{-1}$) of wild-type and arcA mutant (aerobic, anaerobic and nitrate limited) | Toya et al, 2012 |
| Yeast | genome wide | exchange (3) | | Chemostat growth (0.1 $h^{-1}$) using 4 different carbon sources | Lapujade et al, 2004 |
| Yeast | genome wide (relative expression) | internal (83) | metabolomics | Chemostat growth (aerobic, 0.1 $h^{-1}$) of wild-type and gcn4 mutant | Moxley et al, 2009 |
| Yeast | central carbon (41) | exchange (8) | metabolomics | Chemostat growth (anaerobic, 0.03 $h^{-1}$) of 2 yeast strains under glucose and arabinose limited conditions | Wisselink et al, 2010 |
| Yeast | differentially expressed genes (relative expression) | internal (55) | metabolomics | Batch growth (anaerobic) of yeast at different levels of NADPH oxidation | Celton et al, 2012 |
| Yeast | genome wide | exchange (7) | | Chemostat growth (aerobic) at 75% and 85% of maximum growth rate | Lee et al, 2012 |
| Yeast | genome wide | internal (28) exchange (4) | proteomics metabolomics | Chemostat growth (0.1 $h^{-1}$) of yeast at different oxygen levels | Wiebe et al, 2007; Jouhten et al, 2008; Rintala et al, 2009 |

Covert MW, Knight EM, Reed JL, Herrgard MJ, Palsson BO (2004) Integrating high-throughput and computational data elucidates bacterial networks. Nature 429: 92–96.

Fong SS, Nanchen A, Palsson BO, Sauer U (2006) Latent pathway activation and increased pathway capacity enable Escherichia coli adaptation to loss of key metabolic enzymes. Journal of Biological Chemistry 281: 8024–8033.

Ishii N, Nakahigashi K, Baba T, Robert M, Soga T, et al. (2007) Multiple high-throughput analyses monitor the response of E. coli to perturbations. Science Signalling 316: 593.

Holm AK, Blank LM, Oldiges M, Schmid A, Solem C, et al. (2010) Metabolic and transcriptional response to cofactor perturbations in Escherichia coli. Journal of Biological Chemistry 285: 17498– 17506.

Toya Y, Nakahigashi K, Tomita M, Shimizu K (2012) Metabolic regulation analysis of wild-type and arcA mutant Escherichia coli under nitrate conditions using different levels of omics data. Molecular BioSystems 8: 2593–2604.

Daran-Lapujade P, Jansen ML, Daran JM, van Gulik W, de Winde JH, et al. (2004) Role of Transcriptional Regulation in Controlling Fluxes in Central Carbon Metabolism of Saccharomyces cerevisiae, a Chemostat Culture Study. Journal of Biological Chemistry 279: 9125–9138.

Moxley J, Jewett M, Antoniewicz M, Villas-Boas S, Alper H, et al. (2009) Linking high-resolution metabolic flux phenotypes and transcriptional regulation in yeast modulated by the global regulator Gcn4p. Proceedings of the National Academy of Sciences 106: 6477–6482.

Wisselink HW, Cipollina C, Oud B, Crimi B, Heijnen JJ, et al. (2010) Metabolome, transcriptome and metabolic flux analysis of arabinose fermentation by engineered Saccharomyces cerevisiae. Metabolic Engineering 12: 537–551.

Celton M, Sanchez I, Goelzer A, Fromion V, Camarasa C, et al. (2012) A comparative transcrip- tomic, fluxomic and metabolomic analysis of the response of Saccharomyces cerevisiae to increases in NADPH oxidation. BMC Genomics 13: 317.

Lee D, Smallbone K, Dunn W, Murabito E, Winder C, et al. (2012) Improving metabolic flux predictions using absolute gene expression data. BMC Systems Biology 6: 73.

Wiebe MG, Rintala E, Tamminen A, Simolin H, Salusjarvi L, et al. (2008) Central carbon metabolism of Saccharomyces cerevisiae in anaerobic, oxygen-limited and fully aerobic steady- state conditions and following a shift to anaerobic conditions. FEMS Yeast Research 8: 140–154.

Jouhten P, Rintala E, Huuskonen A, Tamminen A, Toivari M, et al. (2008) Oxygen dependence of metabolic fluxes and energy generation of Saccharomyces cerevisiae CEN. PK113-1A. BMC Systems Biology 2: 60.

Rintala E, Toivari M, Pitkanen JP, Wiebe MG, Ruohonen L, et al. (2009) Low oxygen levels as a trigger for enhancement of respiratory metabolism in Saccharomyces cerevisiae. BMC Genomics 10: 461.
